# The association between fetal renal artery indices in late pregnancy and birth weight in gestational diabetes mellitus: A cohort study

**DOI:** 10.18502/ijrm.v20i1.10405

**Published:** 2022-02-18

**Authors:** Ashraf Sadat Jamal, Mahsa Naemi, Laleh Eslamian, Vajiheh Marsoosi, Maryam Moshfeghi, Maryam Nurzadeh, Taraneh Geran, Marjan Ghaemi, Leila Zanbagh

**Affiliations:** ^1^Fetomaternal Department, Shariati Hospital Tehran University of Medical Sciences, Tehran, Iran.; ^2^Department of Endocrinology and Female Infertility, Reproductive Biomedicine Research Center, Royan Institute, Tehran, Iran.; ^3^Department of Obstetrics and Gynecology, Imam Khomeini Hospital Complex, Tehran University of Medical Sciences, Tehran, Iran.; ^4^Vali-e-Asr Reproductive Health Research Center, Tehran University of Medical Sciences, Tehran, Iran.

**Keywords:** Fetus, Gestational diabetes mellitus, Infant, Middle cerebral artery, Renal artery, Doppler ultrasound, Umbilical artery.

## Abstract

**Background:**

Estimation of the fetal birth weight and diagnosis of small for gestational age in the fetuses of women with gestational diabetes mellitus (GDM) are currently imprecise.

**Objective:**

We aimed to evaluate the association between fetal renal artery Doppler indices and neonatal birth weight in women with GDM in late pregnancy.

**Materials and Methods:**

This cohort study recruited 246 pregnant women from Shariati Hospital in Tehran, Iran, in two GDM and healthy control groups. Participants underwent weekly Doppler ultrasounds in the late pregnancy period (37-40 wk) to determine the Doppler indices of the umbilical artery, middle cerebral, and renal arteries. Fetal growth indices including biparietal diameter, abdominal circumference, head circumference, and femur length were also recorded and compared between the two groups.

**Results:**

Fetal growth indices and estimated fetal weight were not significantly different between the two groups. Neonatal birth weight was significantly higher in the GDM group (p < 0.01). The GDM group had significantly higher renal artery indices (resistance index: p = 0.01, pulsatility index [PI]: p = 0.03, and systolic/diastolic ratio [S/D]: p = 0.01) compared to the control group. Also, there was an inverse linear correlation between umbilical indices and birth weight (PI: p = 0.01, S/D: p < 0.01), and between renal artery indices and birth weight (resistance index: p = 0.02, PI: p = 0.01, and S/D: p = 0.03). In the control group, only umbilical artery PI had an inverse linear correlation with birth weight (p = 0.03) and there was no correlation between renal artery indices and birth weight.

**Conclusion:**

Using Doppler hemodynamic indices of the renal artery in late pregnancy in women with GDM can be helpful for early detection of hypoxic fetuses, who are at risk of being small for gestational age or having intrauterine growth restriction, even when of normal weight.

## 1. Introduction

Gestational diabetes mellitus (GDM) is one of the most common endocrine diseases and it affects around 7-10% of all pregnancies worldwide (1). Prevalence rates vary due to the local screening practices and diagnostic criteria as well as population characteristics such as body mass index, maternal age, history of type 2 diabetes, and ethnicity (2). GDM is associated with adverse maternal and neonatal outcomes (3). Doppler vascular ultrasound is a non-invasive method that can evaluate vascular impedance distal to the area.

The placenta transports oxygen and nutrients to the fetus. For evaluation of the fetus blood supply, hemodynamic parameters such as the systolic/diastolic ratio (S/D), the pulsatility index (PI), and the resistance index (RI) play an important role (4). The kidney is one of the main organs of the fetus that is sensitive to hypoxia (5). The resistance of the renal artery may increase in intrauterine growth restriction (IUGR). Therefore, evaluation of the renal artery indices, such as the PI, RI and S/D, provide valuable information on renal vessels. Few studies have focused on fetal renal arteries in women with GDM and there is no consensus on the validity of their parameters (6).

Therefore, the current study sought to compare in late pregnancy, in women with vs. without GDM, the correlations of hemodynamic indices (S/D, PI, and RI) of the umbilical artery, middle cerebral artery and renal artery, and fetal growth with the newborn birth weight. The findings could then be used to determine whether fetal hemodynamic indices can assist clinicians in estimating newborn birth weight and possibly facilitate early detection of hypoxia in fetuses.

## 2. Materials and Methods

### Participants

This cohort study was performed in Shariati Hospital, Tehran University of Medical Sciences, Tehran, Iran from January to December 2020. Two hundred forty-six pregnant women in their late pregnancy period who were referred for ultrasound were divided into two groups. GDM group: the women with GDM, diagnosed by blood test at 24-28 wk of gestational age, treated with insulin or managed with diet; and the control group: women without GDM. The inclusion criteria were women aged 18-40 yr with a singleton viable pregnancy and gestational age from 37 to 40 wk. In the GDM group, women newly identified with GDM according to the 2018 diagnostic criteria of the American Diabetes Association were recruited. GDM was diagnosed by a two-hr oral glucose tolerance test at 24-28 wk of gestational age. A diagnosis was made when any of the following was met or exceeded: fasting glucose level of 92 mg/dl, one-hr level of 180 mg/dl, and/or two-hr level of 153 mg/dl (7). Exclusion criteria in both groups were: multiple pregnancies, pre-gestational diabetes mellitus, preeclampsia (pregnancy-induced hypertension), the use of alcohol/cigarettes, IUGR, or other well-known conditions affecting fetal blood flow. Participants underwent physical examinations and were investigated for fasting blood glucose level, two-hr postprandial blood glucose level, and HbA1C at 37 wk. Participants with a fasting glucose level below 92 mg/dl, an HbA1C below 6, and a 2hPP below 120 mg/dl were defined as having well-controlled GDM. The age, gestational age, mode of delivery, birth weight, Apgar score, umbilical arterial blood pH, neonatal blood glucose level, and neonatal intensive care unit admission were evaluated in both groups.

### Ultrasound assessment

Transabdominal 2D ultrasound was performed for all participants to assess biometry and amniotic fluid index with the exclusion of congenital fetal malformations. Ultrasound assessments were performed by one perinatologist using the Affiniti 70 ultrasound machine (Philips, the Netherlands) and a C6-2 convex probe with a frequency of 2-6 MHz. All recordings were obtained weekly from 37 wk of gestation until delivery, and the last records were evaluated in the analysis. All Doppler measurements were done in the time of lack of fetal movements and breathing (8).

Fetal biometrics, including biparietal diameter, femur length, abdominal circumference, head circumference, and fetal weight, were calculated according to Hadlock's formula (9).

The color flow pattern was selected to measure the RI, PI, and S/D of the umbilical, middle cerebral, and renal arteries. This measurement was performed for the umbilical artery 5 cm apart from the placenta, in the way that the angle between blood flow and the ultrasound beam was below 20°.

For MCA Doppler, the ultrasound probe was located toward the brain basement membrane to see a pair of alisphenoids in the distance of the anterior and middle cranial fossa to reveal the Willis circle (10). The sampling volume (2 mm) was located in the proximal of the third portion of the mentioned artery, just after its origin from the Willis circle (11).

The evaluation of the fetal renal artery was done in the coronal plane near to the renal hilum, and the angle between blood flow and the ultrasound beam was below 20°. The Doppler indices mean of two renal arteries was applied in the final analysis. For the final analysis, the mean of five serial Doppler velocities was used (10).

### Sample size calculation

To obtain a correlation coefficient of at least 0.25 between birth weight and fetal renal artery Doppler hemodynamic indices, with an error of the first type equal to 0.05 and a power of 80%, the required sample size for each group was estimated to be 123.

### Ethical considerations

The study protocol was approved by the Institutional Review Board of Tehran University of Medical Sciences, Teheran, Iran (Code: IR.TUMS.MEDICINE.REC.1399.758). Written informed consent was obtained from all participants before the study. This study was conducted according to the principles of the Helsinki Declaration.

### Statistical analysis

Data were analyzed using the Statistical Package for the Social Sciences (SPSS) version 19.0 (IBM, USA). Continuous data were compared between the two groups with the independent *t* test (if normally distributed) or Mann-Whitney U test (if non-normally distributed). The categorical variables were compared with the Chi-square test. The Pearson or Spearman's rho correlation analysis was carried out to assess the linear relationship between fetal growth and Doppler hemodynamic indices with neonatal birth weight. A two-tailed p < 0.05 was considered significant.

## 3. Results

Table I shows the comparison of maternal, fetal, and obstetric characteristics of the GDM and control groups. Fetal growth indices and estimated fetal weight were not significantly different between the two groups. The mean number of days between the last ultrasound and delivery was four in the GDM group and three in the control group, with a range of zero to seven days and without any significant difference between the groups (p = 0.34).

As seen in table II, the GDM group had a higher median umbilical artery RI, umbilical artery S/D, renal artery RI, renal artery PI and renal artery S/D than the control group.

The mean neonatal birth weight in the GDM group was 3404 gr vs. 3243 gr in the control group (p < 0.01). Ten (8.3%) newborns in the GDM group and 6 (4.9%) in the control group had a birth weight ≥ 4000 gr; this difference was not significant.

Only one neonate of each group had an Apgar score below seven at five min after birth. There was no significant difference between the two groups in terms of umbilical arterial blood pH below 7.2 or neonatal intensive care unit admission. The cesarean section rate was 86.2% in the GDM group vs. 69.9% in the control group (p < 0.01).

In both groups, there was a significant linear correlation between the fetal growth indices and birth weight (p < 0.01) (Table III). The relation between the Doppler indices of the renal and umbilical arteries with neonatal weight is listed in table III.

Fifty-two (41.5%) cases in the GDM group were treated with insulin and 70 (58.5%) were only managing their GM through their diet. None of the cases had metformin or other oral drugs administered. Given the defined criteria for measuring how GM is controlled, 69 (56.0%) cases were well controlled. The proportion of well-controlled cases was significantly different according to their treatment. Twenty-one (41.2%) cases in the insulin subgroup vs. 48 (66.7%) in the diet subgroup were well controlled (p = 0.01). The Doppler indices in the two diabetic subgroups (well controlle d vs. poorly controlled) were not significantly different.

The newborn blood sugar testing revealed five cases (4.1%) of hypoglycemia. Two (3.9%) newborns in the insulin subgroup and three (4.2%) in the diet subgroup were hypoglycemic. Two (2.9%) newborns in the well-controlled GDM subgroup and three (5.6%) in the poorly-controlled GDM subgroup were hypoglycemic; this difference was not significant (p = 0.65). None of the newborns in the control group were hypoglycemic.

**Table 1:** Comparison of maternal, fetal, and obstetric characteristics in the GDM and control groups


**Variables**		**GDM group (n = 123)**	**Control group (n = 123)**	p-value*
**Maternal age (yr) **		31.5 ± 5.4	29.7 ± 5.6	≤ 0.001
**Gestational age at delivery (wk)**		38.6 ± 0.8	38.9 ± 0.8	0.02
**Fetus biometry**				
	**BPD (mm)**	92.3 ± 3.4	92.2 ± 3.4	0.34
	**HC (mm)**	332.1 ± 8.7	332.0 ± 9.6	0.64
	**AC (mm)**	341.4 ± 13.7	338.3 ± 14.5	0.08
	**FL (mm)**	73.6 ± 2.2	73.3 ± 2.5	0.31
	**EFW (gr)**	3341 ± 319	3296 ± 317	0.26
Data are presented as Mean ± SD. GDM: Gestational diabetes mellitus, BPD: Biparietal diameter, HC: Head circumference, AC: Abdominal circumference, FL: Femur length, EFW: Estimated fetal weight, *Independent sample *t* test				

**Table 2:** Comparison of Doppler ultrasound characteristics and pregnancy outcomes in the two study groups


**Variables**	**GDM group (n = 123)**	**Control group (n = 123)**	**p-value**
**UA-RI ${}^{a}$ **	0.59 (0.58 ± 0.08)	0.56 (0.56 ± 0.07)	0.03*
**UA-PI ${}^{a}$ **	0.90 (0.92 ± 0.25)	0.85 (0.88 ± 0.19)	0.19*
**UA-S/D ${}^{a}$ **	2.40 (2.49 ± 0.63)	2.30 (2.35 ± 0.45)	0.03*
**MCA-RI ${}^{a}$ **	0.77 (0.77 ± 0.10)	0.77 (0.77 ± 0.08)	0.94*
**MCA-PI ${}^{a}$ **	1.71 (1.72 ± 0.42)	1.69 (1.73 ± 0.45)	0.72*
**MCA-S/D ${}^{a}$ **	4.40 (4.77 ± 1.81)	4.40 (4.86 ± 2.02)	0.87*
**RA-RI ${}^{a}$ **	0.85 (0.84 ± 0.06)	0.83 (0.83 ± 0.05)	0.00*
**RA-PI ${}^{a}$ **	2.19 (2.21 ± 0.47)	2.05 (2.10 ± 0.44)	0.03*
**RA-S/D ${}^{a}$ **	6.70 (7.25 ± 2.83)	5.80 (6.41 ± 2.27)	≤ 0.001*
**BW (12) ${}^{a}$ **	3404 (3432 ± 387)	3243 (3216 ± 376)	≤ 0.001**
**Macrosomia ${}^{b}$ **	10 (8.3)	6 (4.9)	0.30***
**C/S ${}^{b}$ **	106 (86.2)	86 (69.9)	0.00***
**Umbilical arterial pH < 7.2 ${}^{b}$ **	5 (4.1)	6 (4.9)	0.75***
**NICU admission ${}^{b}$ **	14 (11.4)	16 (13.0)	0.69***
${}^{a}$ Data are presented as Median (Mean ± SD), ${}^{b}$ Data are presented as n (%). UA: Umbilical artery, MCA: Middle cerebral artery, RA: Renal artery, RI: Resistance index, PI: Pulsatility index, S/D: Systolic/diastolic ratio, BW: Birth weight, C/S: Cesarean section, NICU: Neonatal intensive care unit, GDM: Gestational diabetes mellitus, *Mann-Whitney U test, **Independent two sample *t* test, ***Chi-square test			

**Table 3:** The correlation of fetal growth and hemodynamic indices with birth weight in each group


** Indices**	**GDM group r (p-value)**	**Control group r (p-value)**
**BPD **	0.40 ( ≤ 0.001)	0.38 ( ≤ 0.001)
**HC **	0.37 ( ≤ 0.001)	0.45 ( ≤ 0.001)
**AC **	0.62 ( ≤ 0.001)	0.63 ( ≤ 0.001)
**FL **	0.44 ( ≤ 0.001)	0.37 ( ≤ 0.001)
**UA-RI **	-0.17 (0.05)	-0.14 (0.05)
**UA-PI **	-0.22 (0.01)	-0.19 (0.02)
**UA-S/D **	-0.25 (0.00)	-0.16 (0.07)
**MCA-RI**	-0.01 (0.84)	-0.14 (0.10)
**MCA-PI**	-0.06 (0.46)	-0.15 (0.09)
**MCA-S/D**	-0.05 (0.53)	-0.14 (0.10)
**RA-RI **	-0.21 (0.01)	-0.07 (0.40)
**RA-PI **	-0.23 (0.01)	-0.10 (0.23)
**RA-S/D **	-0.20 (0.02)	-0.05 (0.52)
BPD: Biparietal diameter, HC: Head circumference, AC: Abdominal circumference, FL: Femur length, UA: Umbilical artery, MCA: Middle cerebral artery, RA: Renal artery, RI: Resistance index, PI: Pulsatility index, S/D: Systolic/diastolic ratio, GDM: Gestational diabetes mellitus. Pearson (for fetal growth indices) or Spearman's rho (for fetal hemodynamic indices) Correlation analysis		

## 4. Discussion

In our study, impaired Doppler indices in the GDM group were associated with lower birth weight, and these symptoms were first observed in renal artery indices. Fetal weight is not a good predictor of being small for gestational age (SGA) or IUGR in GDM, and biometric factors are not valid either. Hypoxia and SGA or IUGR may occur in GDM mothers, even in a fetus with a weight above the 10 ${}^{\mathrm{th}}$ percentile for their gestational age. Gestational diabetes is associated with some fetal and maternal complications,such as preeclampsia, fetal death or malformation. Indeed, between 20-40% of fetuses are macrosomic in GDM. Increased glycemic indices in GDM can lead to fetal growth abnormalities especially in late pregnancy (13).

Ultrasonography is a non-invasive tool with reproducibility that is cost-effective. It is the optimum method to screen fetal wellbeing. The umbilical and middle cerebral artery as well as the renal artery are the main arteries of the circulatory system of the fetus. Their indices may be a good indicator for fetal growth and predictors of fetal distress, IUGR, and other poor fetal or maternal outcomes.

The renal artery, when faced with hypoxia and ischemia, redistributes blood flow. To ensure an adequate blood supply to the main organs such as the brain and liver, its S/D, PI, and RI increase. We did not expect to see such redistribution in healthy fetuses. In this study, no significant correlation was observed between renal artery indices and birth weight in the control group, which is similar to the results of another previous study (10). As we excluded women with well-known conditions affecting fetal blood flow such as IUGR and hypoxemia in both groups, we did not expect any disturbance in renal artery Doppler indices in the control group. In the GDM group, lower birth weight was assumed to be related to an early hypoxic state in the fetus. A significant correlation was found between blood oxygen deficit and increased renal artery PI (14). A limitation of our study was that we did not directly assess fetal hypoxia, as doing so requires invasive testing.

We found that the hemodynamic indices of the fetal renal artery in term pregnancy (37-40 wk) were inversely correlated with neonatal birth weight in the GDM group. This is contrary to another study that found a statistically significant positive correlation between birth weight and renal artery RI, PI, and S/D in women with GDM (all p < 0.01). This may be because the criteria used for diagnosing diabetes in pregnancy were different.

With increasing gestational age, placental blood is supplemented, the blood volume of the umbilical artery rises, and vascular resistance reduces. In the GDM group, there was a significant negative correlation between newborn birth weight and indices of the umbilical artery (S/D and PI), but we did not detect a significant correlation with RI. This may be because PI is often a more sensitive item compared to RI. In one study, a significant correlation was found between birth weight and umbilical artery PI, but not with the values of the uterine artery PI, which is consistent with the current study (15). In another study on pregnant women with type 1 diabetes, macrosomic fetuses had a significant decline in umbilical artery PI compared with normal-weight fetuses. Their findings demonstrated a negative correlation between umbilical PI and neonatal birth weight (16). A further study also showed that there can be a negative correlation between umbilical PI and fetal weight which is in line with the current study (17). And a recent study of 226 women with GDM revealed that the umbilical artery hemodynamic indices in late pregnancy had a negative correlation with neonatal birth weight, but there was no such correlation with fetal growth indices (18).

If blood demand increases, the fetus may be at risk for hypoxia. In such cases, blood flow is altered to spare the brain, which leads to an increase in MCA blood supply, providing 80% of cerebral hemisphere perfusion (19). However, we did not find any differences in the middle cerebral artery indices between the groups as we excluded cases of IUGR from our study.

## 5. Conclusion

Our study showed that in mothers with GDM, the hemodynamic indices of the fetal renal artery in term pregnancies (37-40 wk) were inversely correlated with neonatal birth weight. Based on our findings, we propose that the use of Doppler hemodynamic indices of the renal artery in late pregnancy can be a helpful parameter in GDM mothers to detect hypoxic fetuses who are at risk of SGA and IUGR even with a normal weight (above the 10 ${}^{\mathrm{th}}$ percentile for their gestational age). These renal artery Doppler indices can help diagnose hypoxia in these fetuses even earlier than the umbilical artery Doppler indices.

##  Conflict of Interest

The authors declare that there is no conflict of interest.
